# The effect of selenium therapy in critically ill patients: an umbrella review of systematic reviews and meta-analysis of randomized controlled trials

**DOI:** 10.1186/s40001-023-01075-w

**Published:** 2023-02-28

**Authors:** Salman Jaff, Sheida Zeraattalab-Motlagh, Reza Amiri Khosroshahi, Mohammed Gubari, Hamed Mohammadi, Kurosh Djafarian

**Affiliations:** 1 Department of Clinical Nutrition, School of Nutritional Sciences and Dietetics, Tehran University of Medical Sciences, P.O. Box 14155-6117, Tehran, Iran; 2 Department of Community Nutrition, School of Nutritional Sciences and Dietetics, Tehran University of Medical Sciences (TUMS), Tehran, Iran; 3 Department of Community and Family Medicine, School of Medicine, University of Sulaimani, Sulaymaniyah, Iraq

**Keywords:** Selenium, Meta-analysis, Mortality, Supplementation, Randomized controlled trials

## Abstract

**Background:**

Selenium is an essential nutrient with antioxidant, anti-inflammatory, and immuno-regulatory properties. Studies have displayed that in critically ill patients, selenium supplementation may be a potentially promising adjunctive therapy.

**Objective:**

We aimed to present an overview of the effects of selenium supplementation in adult critically ill patients based on published systematic reviews and meta-analyses (SRMAs) of randomized controlled trials (RCTs).

**Methods:**

A literature search in three electronic databases, PubMed, Scopus, and Web of Science, was performed to find eligible SRMAs until July 2022. For each outcome, the risk ratios (RRs) or mean differences (MDs) and 95% confidence intervals (CIs) were recalculated using either random or fixed effect models. The methodological quality and quality of evidence of the SRMAs were assessed by applying “A Measurement Tool to Assess Systematic Reviews” (AMSTAR2) and Grading of Recommendations Assessment, Development, and Evaluation(GRADE) tools, respectively.

**Results:**

We included 17 meta-analyses containing 24 RCTs based on inclusion criteria. Selenium supplementation can reduce the incidence of mortality (RR: 0.83, 95% CI 0.71, 0.98, *P* = 0.024) and incidence of acute renal failure (RR: 0.67, 95% CI 0.46, 0.98, *P*: 0.038) significantly; however, the certainty of evidence was low. Moreover, with moderate to very low certainty of evidence, no significant effects were found for risk of infection (RR: 0.92, 95% CI 0.80, 1.05, *P*: 0.207), pneumonia (RR: 1.11, 95% CI 0.72, 1.72, *P*: 0.675), as well as the length of ICU (MD: 0.15, 95% CI − 1.75, 2.05, *P*: 0.876) and hospital stay (MD: − 0.51, 95% CI − 3.74, 2.72, *P*: 0.757) and days on ventilation (MD: − 0.98, 95% CI − 2.93, 0.98, *P*: 0.329).

**Conclusions:**

With low quality of evidence, the use of selenium supplementation could improve the risk of mortality and acute renal failure, but not other outcomes in critically ill patients.

**Supplementary Information:**

The online version contains supplementary material available at 10.1186/s40001-023-01075-w.

## Introduction

Critical illness is a stress condition that activates the oxidant network. In critically ill patients, oxidative stress plays a pivotal role in pathophysiological events resulting in mitochondrial dysfunction and systemic inflammatory response syndrome (SIRS), which may be complicated and lead to acute respiratory distress syndrome and multiple organ dysfunction [1]. Oxidative stress is defined as a state in which the levels of toxic reactive oxygen species (ROS) overcome the endogenous antioxidant defenses [2] and can be due to either extra oxidant production or antioxidant defense depletion [3].

The endogenous antioxidant defense systems are remarkably effective at neutralizing ROS and other reactive species [4]. These systems include antioxidant enzymes (superoxide dismutase, glutathione peroxidase, and catalase) and their cofactors (vitamin C, E, β-carotene, zinc, copper, manganese, and selenium) [5]. Selenium is a critical factor in controlling immunity and inflammation responses and a necessary micronutrient for more than 25 proteins in the body. These proteins have several functions, containing antioxidant defense, protein folding [6], and thyroid hormone metabolism [7]. Documents report that selenium is a vital trace element in the antioxidation process [8–10]. Moreover, selenium deficit is often recognized in patients with sepsis, especially those with poor quality diets, chronic disease, gastrointestinal illness, and critical illness [11, 12].

Critical illness is associated with decreased supplies of antioxidants, decreased plasma or intracellular concentrations of free electron scavengers or cofactors, and reduced activities of enzymatic systems involved in ROS detoxification [5, 13, 14]. Evidence suggests that in critically ill patients, plasma selenium is significantly under the normal range. Also, it has been indicated that depletion of this micronutrient is associated with a worse clinical outcome, such that low selenium levels were related to more infectious complications and a higher incidence of mortality [15]. Although several systematic reviews have been published about the effectiveness of selenium supplementation in critically ill patients, the evidence is unclear. For instance, Huang et al. [16], in a systematic review that analyzed data from seven randomized controlled trials (RCTs), indicated the positive effect of selenium supplementation on decreasing the length of hospital and intensive care unit (ICU) stay and mortality in trauma patients. However, Manzanares et al. [17], in a systematic review that analyzed data from 21 RCTs, showed that supplementation with selenium had no significant impact on the reduction of mortality and ameliorated other clinical outcomes in critically ill patients. Besides, previous studies did not examine the strength of the quality of evidence in total. Therefore, due to these inconstancies and uncertainties, this umbrella review of systemic reviews and meta-analyses (SRMAs) aimed to identify the benefits of selenium supplementation compared to any control groups in adult critically ill patients.

## Methods

### Registration and reporting format

This umbrella review was conducted by the guidance outlined in the Cochrane Handbook for Systematic Reviews of Interventional trials [18] and the Grading of Recommendations, Assessment, Development, and Evaluation (GRADE) approach [19], and it was outlined in accordance with ‘The Preferred Reporting Items for Overviews of Reviews’ (PRIOR) framework [20] The protocol of this review was registered in the PROSPERO international prospective register of systematic reviews (ID number: CRD42022347493).

### Search strategy

Until July 2022, two authors (SJ and RA) systematically searched three electronic databases, including MEDLINE via PubMed, Scopus, and ISI Web of Science, for eligible SRMAs that evaluated the effects of selenium therapy among critically ill patients (Additional file 1: Table S1). Reference lists of retrieved published articles were hand searched to identify relevant SRMAs not identified through electronic searches. Any discrepancies were resolved by discussion and consultation with the third author (KD).

### Eligibility criteria

Articles were considered eligible if [1] SRMAs of RCTs were conducted in critically ill adults (≥ 18 years old); (2) had parenteral selenium supplementation in the intervention group (with/without beginning with bolus) as a monotherapy compared with any control group (including, placebo, no treatment, normal saline standard care, and a low dose of selenium); (3) systematic reviews that have performed analysis and reported the risk ratio (RR) or mean difference (MD) with a 95% confidence interval (CI) for the effect of selenium therapy on total and 28 days mortality, infection, adverse events, pneumonia, acute renal failure, length of hospital stay, length of ICU stay, and days on ventilation. Systematic reviews without meta-analysis and if the study was conducted on children, pregnant, or lactate women were excluded. We chose RCTs that were included in the reviews. When more than one study reported data for the same outcome, the article with further complete information was selected. Two reviewers (SJ and RA) screened the title and abstract and eligible studies were selected. Then, the same pairs of reviewers critically appraised the full text of selected studies. Any disagreement was addressed through discussion with the third author (KD) until agreements were obtained.

### Data extraction

The data extraction was done independently by two authors (SJ and SZM). The outlined data was extracted from the selected SRMAs: the name of the first author, year of publication, the number of cases and participants in each study arm (intervention and control), age and sex of participants, study design, type of selenium supplementation, time of intervention, follow-up time, outcomes of each study, effect sizes (RR or odds ratio (OR) or MD), and corresponding 95% CIs. These items were extracted from the RCTs: name of the first author, year of publication, country, the study design, number of participants in the intervention and control groups, Acute Physiology and Chronic Health Evaluation (APACHE), Sequential Organ Failure Assessment (SOFA), and Simplified Acute Physiology (SAPS) scores, type of selenium in the intervention and control groups, mean age of participants, duration of follow-up, and clinically relevant outcomes.

### Assessment of methodological quality

The methodological quality of included articles was evaluated by the ‘A Measurement Tool to Assess Systematic Reviews’ Version 2.0 (AMSTAR2), a reliable strategy for assessing the quality of systematic reviews and meta-analyses [21]. We also used the Cochrane tool to appraise the risk of bias (ROB) of the RCTs included in each meta-analysis [22]. Assessments were done by two authors (RA and SZM), and any disagreements were addressed by consensus.

### Data synthesis and analysis

We selected primary trials from eligible SRMAs and then added other trials not included in the largest meta-analysis. We took data from the meta-analyses and regenerated RR and MD by applying DerSimonian and Laird random-effects model [23] and using the fixed effect model [24]. For six outcomes (mortality, 28-day mortality, infection, adverse events, pneumonia, and acute renal failure), the pooled RR and 95% CI were estimated, and for the remaining three outcomes (length of ICU stay, length of hospital stay, and days on ventilation), the MD and its 95% CI were recalculated. *I*^2^ statistic was used to estimate the heterogeneity between studies [25]. The *I*^2^ values were interpreted as follows by the Cochrane Handbook guidance: 0%–40% may be not important, 30%–60% may be represented as direct heterogeneity, 50%–90%: can appear as considerable heterogeneity, and 75%–100%: considerable heterogeneity [18]. To evaluate the source of heterogeneity, we performed a subgroup analysis based on the first dose (≤ 1000 and > 1000 µg/day), following dose (≤ 1000 and > 1000 µg/day), dose of selenium in the control group (low dose or not), and duration of follow-up (≤ 10 and > 10 days). An estimate of publication bias in each meta-analysis was presented as a result of Egger’s regression test [26]. Statistical analyses were conducted using STATA software, version 14.0 (StataCorp). *P* < 0.05 was considered significant statistically.

### Grading of the evidence

We assessed the strength of evidence for each outcome presented in the umbrella review through the GRADE approach [27] and classified evidence into “high,” “moderate,” “low,” and “very low” quality. High grades demonstrate high certainty that the true effect is proportionate to the estimate of the effect. A moderate grade indicates that the true effect is likely near the estimated effect; however, there is a slight probability of substantial differences. Low grades suggest a greater likelihood that the actual effect is possibly extensively different from the estimate of the effect, and very low grades indicate that the real effect is probably, different from the estimated effect [28]. Study limitations may include downgraded RCTs with an initial high-quality evidence evaluation. Limitations also include the risk of bias, inconsistency (i.e., remarkable unexplained heterogeneity, *I*^2^ > 50%; *P* < 0.05), indirectness of outcomes (i.e., primarily presented outcomes have been replaced by important patient outcomes) [29], imprecision (i.e., 95% CI for estimated effect is broad or overlaps with the minimal clinical important difference (MCID)), and further considerations (publication bias and dose–response gradient use). In the insufficient evidence outcomes in the literature, MCID was determined in such a way that baseline standard deviations (SDs) were calculated for each outcome from primary trials included in the analysis, and the MCID was defined as half of the SD change in that outcome [30].

## Results

### Study selection

**Fig. 1:**
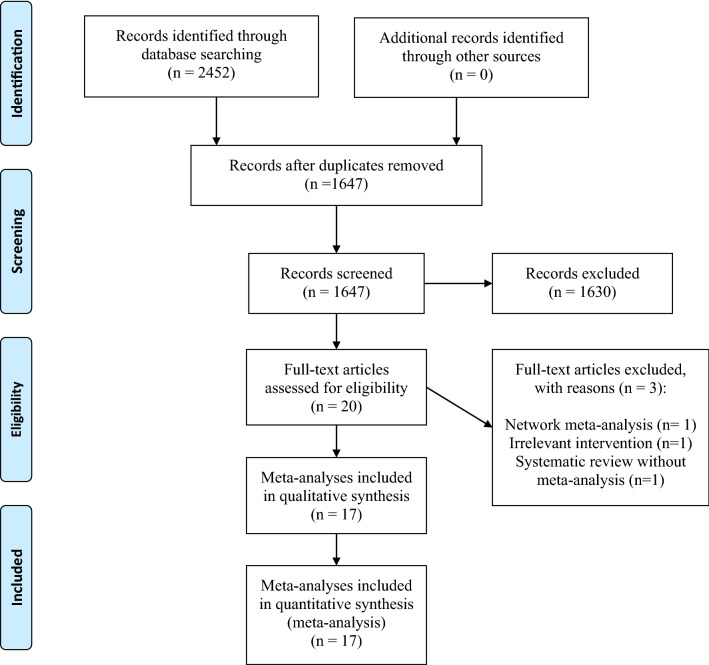
Literature search and study selection process

The detailed process of systematic search and selection of eligible studies is presented in Fig. 1. We searched 2472 articles from electronic databases: PubMed, Scopus, and ISI Web of Science. After duplicate removal, 1647 reviews were considered. Following title and abstract screening, 20 records remained eligible for full-text assessment. The excluded studies and the reason for exclusion are presented in Additional file 1: Table S2.

Finally, 23 RCTs from 17 SRMAs [5, 16, 17, 31–44] examined the efficacy of selenium supplementation compared to any control. Nine critical illness-related outcomes, including mortality, 28-day mortality, infection, adverse events, pneumonia, acute renal failure, length of ICU stay, length of hospital stay, and days on ventilation, were extracted from all eligible studies. The populations included patients with gastrointestinal bleeding, SIRS, sepsis, septic shock, trauma, severe septic shock with documented infection, mechanical ventilation, acute pancreatic necrosis, acute respiratory distress syndrome*,* cardiac arrest after cardiopulmonary resuscitation, and elective cardiac surgery.

### Study characteristics of RCTs from the systematic reviews and meta-analyses

The publication years of the included studies were recorded from 1983 to 2018. The amounts of selenium varied from 474 to 4000 μg/day at the first dose and up to 1600 μg/day at the following dose. The follow-up duration ranged from 1 to 52 weeks. In the intervention groups, selenium and sodium selenite were used. Control groups used low doses of selenium or sodium selenite (standard treatment) and normal saline as a placebo. Two studies had no treatment, and one did not mention the treatment in the control groups (Table 1).

**Table 1:** Study characteristics of randomized controlled trials from eligible systematic reviews and meta-analyses

First author, year, country (ref)	Study design	Sample size, intervention/placebo, Mean age, critical illness types	Intervention (s): first and following dose	Comparison (s)	APACHE or SAPS or SOFA score, intervention/placebo	Follow-up duration	Outcomes
Andrew, 2011, UK [45]	A double-blinded randomized parallel trial	127/125, 64.5 years, GI bleeding	Intravenous sodium selenite 500 μg/day for 7 day	Selenium ≤ 50 μg /day	APACHE II: 20/20	14 day	Infection, adverse events, LOS hospital, LOS ICU, mortality by duration (28 days), mortality
Angstwurm, 2007, Germany [46]	Prospective randomized, placebo-controlled, multiple-center trial	122/124, 64.6 years, SIRS, sepsis, and septic shock	Bolus and continuous intravenous sodium selenite bolus 1000 μg/day, followed by a continuous dose of 1000 μg/day	Placebo	APACHE III: 92.2/91.2	28 days	Mortality, mortality by duration (28 days), infection, adverse events, pneumonia, LOS ICU
Angstwurm, 1999, Germany [47]	Prospective open-label pilot RCT	21/21, 56 years, SIRS	Continuous intravenous sodium selenite, 535 μg/day for 3 days then 285 μg/day for 3 days then 155 μg/day for 3 days then 35 μg/day for the remainder of the total treatment time	Placebo	APACHE II: ≥ 15/≥ 15	Until discharge	Adverse event, LOS hospital, LOS ICU, renal failure length of ventilation, mortality by duration (28 days), mortality
Bloos, 2016, Germany [48]	Placebo-controlled trial, multi-center	543/546, 65.7 years, mixed ICU patients with severe sepsis or septic shock in last 24 h	Sodium selenite, IV loading dose of 1000 μg/day followed by a continuous 1000 μg/day	Placebo	APACHE II: 24.7/24.4	90 days	Infection, adverse events, LOS ICU, mortality by duration (28 days), mortality
Brodska, 2015, Czech Republic [60]	Prospective, randomized, open-label single center	75/75, 60 years, SIRS/sepsis	Intravenous sodium selenite, 1000 μg/day on the first day, 500 μg/day on subsequent days in the form of Na selenite pentahydrate	Standard selenium dose (< 75 μg/day)	APACHE II: 30/28	Until discharge	Mortality by duration (28 days)
Berger, 2001, Switzerland [7]	Prospective couple blind RCT	9/12, 42 years trauma patients	Slow intravenous selenium 500 μg/day	Placebo	APACHE II: 13/11	20 days	Infection, LOS ICU, days on ventilation, mortality by duration (28 days), mortality
Chelkeba, 2017, Iran [49]	Prospective RCT	29/25, 38 years, septic patients	Intravenous selenium selenite, high‑dose parenteral sodium selenite (2 μg/day intravenous bolus, followed by 1.5 μg/day IV continuous infusion plus standard therapy	Standard therapy	APACHE II: 17/16.4	90 days	LOS ICU, LOS hospital, days on ventilation, renal failure, mortality, mortality by duration (28 days)
Forcevill, 2007, France [50]	Double-blind, phase II RCT	31/29, 67.5 years, severe septic shock patients with documented infection and mechanically ventilated	Intravenous sodium selenite, 400 μg/day (in 192 mL saline) over 24 h, then 1000 μg/da (in 48 mL saline) over 24 h for 9 days	Placebo	NR	208 days	Infection, pneumonia, adverse event, LOS ICU, LOS hospital, days of ventilation, renal failure, mortality by duration (28 days), mortality
Gartner, 1983, Germany [61]	Double-blind parallel RCT	21/21, SIRS/sepsis	Intravenous sodium selenite, 500 μg/day, 250 μg/day, and 125 μg/day, each amount for 3 days	35 μg/day sodium selenite during the whole treatment period	APACHE II: 17/19	48 days	Renal failure, mortality by duration (28 days)
Janka, 2013, Slovak Republic [51]	Prospective observational trial	35/37, 53 years, septic patients	Continuous intravenous sodium selenite pentahydrate at 750 μg/day	Placebo	APACHE II: 24/34	28 days	Mortality by duration (28 days), mortality
Khalili, 2017, Iran [62]	Clinical trial	125/182, 35.5 years, traumatic brain injury	Sodium selenate pentahydrate intravenously, 1000 μg/day for the first 5 days, followed by 500 μg/day for an additional 5 days	No treatment	NR	96 days	Mortality by duration (28 days), LOS hospital, LOS ICU
Kuklinski, 1991, Germany [52]	RCT	8/9, 46.5 years, acute pancreatic necrosis	Intravenous 500 μg/day sodium selenite	No treatment	NR	NR	Mortality, mortality by duration (28 days)
Lindner, 2004, Germany [53]	Prospective randomized trial	35/35, 51 years, severe acute pancreatitis	Intravenous sodium selenite day 1, 2000 μg/day, days 2–5, 1000 μg/day, day 6 until discharged 300 μg/day	Placebo	NR	90 days	Mortality, infection
Mahmoodpoor, 2018, Iran [63]	clinical trial	20/20, 54.7 years, acute respiratory distress syndrome	Intravenous sodium selenite, 3000 μg/day in as an initial bolus during the first 3 h of first day followed by 1500 μg/day at the same hourly interval on days 2–10	Normal saline	APACHE II: 21/23	NR	Mortality by duration (28 days), infection, LOS hospital, days on ventilation
Moghaddam, 2017, Iran [65]	Double-blinded controlled trial	57/56, 41.5 years, traumatic brain injury	Intravenous sodium selenite, 500 μg/day for 30 min and then 500 μg/day se during 24 h continuously	Standard care	APACHE III: 49.91/49.34	32 days	Days of ventilation, LOS ICU, LOS hospital, Mortality by duration (28 days)
Manzanares, 2011, Uruguay [54]	Prospective, placebo-controlled, randomized, single-blinded phase II study	15/16, 58 years, SIRS	Intravenous selenium bolus loading 2000 μg/day then 1600 μg/day for10 days	0.9% NaCl only for 10 days	APACHE II: 21/23	28 days	Mortality, renal failure, days of ventilation, LOS ICU, infection, pneumonia, adverse events
Manzanares, 2010, Uruguay [64]	Prospective, placebo-controlled, randomized, single-blinded phase II study	10/10, 42.5 years, SIRS/sepsis	Intravenous selenium, 2000 μg/day (as selenious acid) bolus loading dose over 2 h thereafter 1600 μg/day for 10 days	Loading dose of selenium selenite 1200 μg over 2 h and thereafter 800 μg as a continuous intravenous infusion	APACHE II: 21/23	NR	Mortality by duration (28 days), infection, renal failure, LOS ICU
Montoya, 2009, Mexico {56]	Prospective, randomized, double-blind trial	34/34, 66 years, sepsis	Selenium 1000 μg/day IV on day 1 then 500 μg/day on day 2 then 200 μg/day for 7 day	100 μg/day selenium up to the end of the trial	APACHE II: 17.6/19.8	NR	Mortality, LOS hospital, days of ventilation
Mishra, 2007, UK [55]	Double-blind controlled trial	18/22, 66 years, sepsis	Intravenous selenium, 470 μg/day for 3 days, then 320 μg/day for 3 days, then 160 μg/day for 3 days, and 30 μg/day thereafter	30 μg/day selenium	APACHE II: 17.6/19.8	28 days	Mortality, mortality by duration (28 days), adverse events, renal failure, LOS ICU
Reisinger, 2009, Austria [66]	Double-blind, placebo-controlled, randomized trial	124/102, 65 years, cardiac arrest after CPR	Intravenous sodium selenite, infusion of 200 to 1000 μg/day over 30 min	Placebo	SAPSIII:59/59	96 days	Mortality by duration (28 days)
Schmidt, 2018, Switzerland [67]	Randomized,placebo-controlled, double-blinded clinical trial	206/205, 67 years, elective cardiac surgery	Intravenous bolus of 4000 μg/day sodium selenite and then 1000 μg/day	Placebo	SAPSII: 28/29	NR	LOS ICU, LOS hospital, renal failure, mortality by duration (28 days)
Valenta, 2011, Czech Republic [57]	Prospective randomized, open-label, single-center clinical trial	93/89, 28 years, SIRS/sepsis	Na selenite, 1000 μg/day on the first day, 500 μg/day on days 2–14 via a central venous catheter	Standard Selenium dose (< 75 μg/day)	APACHE II: 30/28	28 days	Mortality, mortality by duration (28 days)
Woth, 2014, Hungary [58]	RCT	23/17, 20 years, severe septic	Intravenous sodium selenite, bolus for 30 min on the first day, the treatment group received 1000 µg/day continuous infusion for 14 days	NR	SOFA score > 2	7 days	Mortality, mortality by duration (28 days)
Zimmerman, 1997, Germany [59]	A prospective randomized study	20/20, SIRS	Intravenous Na-Selenite, 1000 μg/day bolus then 1000 μg/day as a continuous infusion	Standard therapy	APACHE II: 15/15	28 days	Mortality, mortality by duration (28 days)

*APACHE* Acute Physiology and Chronic Health Evaluation, *CPR* cardiopulmonary resuscitation, *IV* intravenous, *ICU* intensive clinical care, *LOS* length of stay, *NR* not reported, *RCT* randomized controlled trial, *SAPS* Simplified Acute Physiology Score, *SIRS* systemic inflammatory response syndrome, *SOFA* Sequential Organ Failure Assessment score

### Methodological quality of the systematic reviews and meta-analyses

The methodological quality of the included meta-analyses was evaluated using the AMSTAR2 tool [21]. The main issue with the included SRMAs was that the majority of them did not fully explain the included trials [5, 17, 31, 34, 36, 39, 40, 42] and did not register the protocol before conducting the meta-analysis [17, 31, 34, 39–42, 44]. Four (23.5%) [32, 33, 35, 43], one (5.9%) [5], six (35.3%) [16, 17, 31, 36–38], and six (35.3%) [34, 39–42, 44] SRMAs were identified to have a high, moderate, low, and critically low quality, respectively (Additional file 1: Table S3).

## Findings from the meta-analysis

### Primary outcomes

#### Mortality (regardless of the period of time)

In total, 16 primary studies [7, 45–59] from five meta-analyses [5, 17, 32, 35, 36] assessed the effect of selenium therapy on overall mortality. Compared to a control group, selenium significantly decreased the incidence of mortality independent of the period of time (RR: 0.83, 95% CI 0.71, 0.98, *P* = 0.024 *I*^2^: 31.6%, GRADE = low certainty) (Additional file 1: Figure S1, Table S4 and Table 2). Our result remained significant in the subgroup analysis based on pre-defined variables, as in the following dosage over 1000 μg/day and in the subgroup of trials that did not contain selenium in the control group (Additional file 1: Table S5). Egger’s test revealed a publication bias that was statistically significant (*P* = 0.036). So, to identify the bias’s source, we applied the trim-and-fill method, but the results remained the same.

**Table 2:** The effect of selenium therapy in critically ill patients

Outcomes	Comparison (s)	Number of trials	Number of participants (intervention/control)	Follow-up (range), wk	Dose (range) µg/day	Effect size (95%CI)	Absolute effect (95% CI)	*P*-value	*I*^2^ (%)	*P*_heterogeneity_	Egger’s test	Certainty of evidence (GRADE)
Mortality (regardless of period of time)	Placebo, no treatment, standard care and low dose of selenium	16	2324 (1161/1163)	1–52	30–4000	RR, 0.83 (0.71, 0.98)	14 lower (24 lower to 3 lower)	**0.024**	31.6	0.110	**0.036**	Low
Mortality by duration (28 days)	Placebo, no treatment, standard care, normal saline and low dose of selenium	21	3714 (1839/1875)	1–52	30–4000	RR, 0.95 (0.82, 1.09)	10 lower (16 lower to 4 lower)	0.478	21.1	0.189	0.476	Moderate
Risk of acute renal failure	Placebo, standard care, low dose of selenium	8	700 (359/341)	1.5–2	30–4000	RR, 0.67 (0.46, 0.98)	5 lower (10 lower to 0)	**0.038**	0.0	0.659	0.180	Low
Adverse events	Placebo and low dose of selenium	7	2014 (1002/ 1012)	1–3	30–4000	RR, 0.98 (0.88, 1.10)	51 lower (79 lower to 23 lower)	0.788	26.4	0.227	0.761	Moderate
Risk of infection	Placebo, normal saline and low dose of selenium	9	2083 (1037/1046)	2–52	300–4000	RR, 0.92 (0.80, 1.05)	0.0 (3 lower to 4 upper)	0.207	0.0	0.620	0.673	Moderate
Risk of pneumonia	Placebo	3	280 (138/142)	4–52	1000–4000	RR, 1.11 (0.72, 1.72)	3 lower (17 lower to 11 uppers)	0.625	5.1	0.349	0.117	Low
Length of ICU stay	Placebo, no treatment, standard care, low dose of selenium	13	2928 (1431/1497)	2–52	30–4000	MD, 0.15 (− 1.75, 2.05)	–	0.876	95.8	< 0.001	0.850	Very low
Length of hospital stay	Placebo, no treatment, normal saline standard care, low dose of selenium	9	1597 (774/823)	2–52	35–3000	MD, − 0.51 (− 3.74, 2.72)	–	0.757	96.0	< 0.001	0.428	Very low
Days on ventilation	Placebo, normal saline standard care, low dose of selenium	8	429 (216/213)	3–52	35–4000	MD, − 0.98 (− 2.93, 0.98)	–	0.329	76.4	< 0.001	0.186	Very low

*ARF* acute renal failure, *CI* confidence interval, *GRADE* Grading of Recommendations Assessment, Development, and Evaluation, *ICU* intensive care unit, *MD* mean difference, *RR* risk ratio, *wk* week

Bold values indicate statistical significance at the P < 0.05

#### Mortality by duration (28 days)

The effect of selenium therapy on 28-day mortality was evaluated in 21 RCTs [7, 45–52, 55, 57–67] from five meta-analyses [32, 36, 38, 41, 44]. In comparison to the control group, critically ill patients who received intravenous selenium had a lower 28-day mortality rate, although this difference was not statistically significant (RR: 0.95, 95% CI 0.82, 1.09, *P*: 0.478, *I*^2^: 21.1%, GRADE = moderate certainty) (Additional file 1: Figure S2, Table S4 and Table 2). The result did not vary from the main analysis in any of the groups in the subgroup analysis based on pre-defined variables (Additional file 1: Table S5). Egger’s test revealed no indication of publication bias (*P* = 0.476).

### Secondary outcomes

#### Risk of acute renal failure

Patients’ incidence of acute renal failure was reported in eight primary trials [47, 49, 50, 54, 55, 61, 64, 67] of three SRMAs [36, 41, 44]. High dosages of selenium administration significantly decreased the incidence of acute renal failure when compared to the control group (RR: 0.67, 95% CI 0.46, 0.98, *P*: 0.038, *I*^2^: 0.0%, GRADE = low certainty) (Additional file 1: Figure S3, Table S4 and Table 2). In the subgroups, only patients who received a dose ≤ 1000 µg/day had a significantly lower occurrence of acute renal failure than the control group (RR: 0.57, 95% CI 0.35, 0.94). In the rest of the subgroups, despite the reduction of patients with acute renal failure with selenium intervention, it was not statistically significant (Additional file 1: Table S5). No evidence of publication bias was found using Egger’s test (*P* = 0.180).

#### Other secondary outcomes

No statistically significant beneficial effects were found for risk of infection (RR: 0.92, 95% CI 0.80, 1.05, *P*: 0.207, GRADE = moderate certainty) and pneumonia (RR: 1.11, 95% CI 0.72, 1.72, *P*: 0.675, GRADE = low certainty), as well as the length of ICU (MD: 0.15, 95% CI − 1.75, 2.05, *P*: 0.876, GRADE = very low certainty) and hospital stay (MD: − 0.51, 95% CI − 3.74, 2.72, *P*: 0.757, GRADE = very low certainty) and days on ventilation (MD: − 0.98, 95% CI − 2.93, 0.98, *P*: 0.329, GRADE = very low certainty) (Additional file 1: Figures S4–S8, Table S4 and Table 2). Among these outcomes, there was considerable heterogeneity for the outcomes of length of hospital and ICU stay and days on ventilation due to *I*^2^ above 75%. Using subgroup analysis based on pre-defined variables, there was no significant difference between stratifications, and the source of heterogeneity was not detected (Additional file 1: Tables S5, S6). Egger’s test did not reveal any significance for any of these outcomes, and thus we did not have a publication bias.

#### Adverse events

Adverse events were evaluated and recorded in seven primary studies [15, 45–48, 54, 55] out of three meta-analyses [32, 35, 36]. The incidence of adverse events in the intervention group was lower than that of the control group with a moderate certainty of evidence, while this difference was not statistically significant (RR: 0.98, 95% CI 0.88, 1.10, *P*: 0.788, *I*^2^: 26.4%, GRADE = moderate certainty) (Additional file 1: Figure S9, Table S4 and Table 2). *I*^2^ (26.4%) detected in this outcome revealed that there was no considerable heterogeneity between the trials. According to the results of the subgroup analysis, the incidence of adverse events was higher compared to the control group in patients who took selenium at initial doses above 1000 µg/day (RR: 1.19, 95% CI 0.95, 1.49), subsequent doses above 1000 µg/day (RR: 1.11, 95% CI 0.82, 1.50), in studies where selenium was based on the control group (RR: 1.08, 95% CI 0.64, 1.82), and where the intervention lasted less than 10 days (RR: 1.11, 95% CI 0.82, 1.49); however, these differences were not statistically significant (Additional file 1: Table S5). Using Egger’s test, no indication of publication bias was discovered (*P* = 0.761).

## Discussion

This is the first and most comprehensive umbrella review that critically reviews the effect of selenium supplementation among critically ill patients. We aimed to simplify knowledgeable clinical decisions when critically ill patients consider selenium supplementation for improving their illness-related outcomes. Overall, there was a low certainty of evidence that selenium had a statistically significant effect on the improving risk of overall mortality and acute renal failure compared with any control (placebo, no treatment, standard care, and a low dose of selenium). However, with moderate to very low quality, evidence on the usage of selenium for improving the risk of 28-day mortality, adverse event, infection, and pneumonia, as well as the length of ICU and hospital stay and days on ventilation, was not statistically significant.

Our findings revealed that selenium supplementation might be helpful in the reduction of the risk of mortality. Moreover, in the subgroup analysis, we found that selenium supplementation in the  studies with  dosage of over 1000 µg/day and in the trials that did not contain selenium in the control group significantly reduced mortality. Similar to our findings, several reviews confirmed that selenium supplementation led to a reduction in the risk of mortality in critically ill patients [16, 31, 34–36, 39, 43]. However, other reviews did not observe a significant effect of selenium supplementation on reducing mortality in critically ill patients [17, 32, 33, 37, 38].

Decreasing selenium plasma levels in critically ill patients was inversely associated with mortality [68]. This hypothesis indicated that selenium supplementation could ameliorate clinical outcomes because of the influence of selenium on the cellular immune function, and it is a necessary cofactor in glutathione enzymatic function [69]. Mitochondrial dysfunction, SIRS, and the multiple organ dysfunction syndrome in critical illness are caused by a notable redox imbalance [1, 70]. Selenium has antioxidant, anti-inflammatory, and immunomodulatory effects [71], and has been displayed to be able to produce inhibition of nuclear factor kappa-B (NF-kB) binding to DNA by regulating gene expression of selenoprotein [72, 73]. It is also likely capable of inducing apoptosis and cytotoxicity in activated proinflammatory cells [74]. In addition, selenium represses C-reactive protein synthesis and increases L-selectin release from monocytes, while diminishing soluble L-selectin, which has been declared in septic patients to be related to elevated mortality [75].

Our results suggested that the effects of selenium supplementation on mortality may be due to the inhibition of chain reactions that are the basis for the development of mitochondrial dysfunction, SIRS, and organ failure [39].

We also revealed a favorable effect of selenium supplementation on acute renal failure. Our subgroup analysis indicated that patients who received a dose of selenium supplementation ≤ 1000 µg/day had a remarkably lower incidence of acute renal failure than the control group. In contrast to our findings, most SRMAs did not show any significant effect on the incidence of acute renal failure [17, 36, 38, 44]. Only Mousavi et al. obtained similar results to our study and found that parenteral selenium supplementation at the first and following doses lower than 1000 mg reduced the risk of acute renal failure by 76% and 47% [41].

Our discrepancy with previous reviews might be due to the following reason. Our study included 24 trials, 8 of which examined the effect of selenium supplementation on acute renal failure in critically ill patients, while the meta-analysis by Landucci et al. [44] included 9 trials, 6 of which had small sample sizes (*n* ˂ 100), and only 4 trials assessed renal failure. In Kong et al.’s meta-analysis [36], only two of their five trials examined acute renal failure, and Li et al.’s study [38] contained five trials on the effect of selenium supplementation on acute renal failure that was restricted to sepsis patients. It seemed that the small number of participants and trials showed a limitation on the importance of the results and their quality.

The underlying mechanism for the significant impact of selenium on the reduction of the risk of acute renal failure may be as follows: oxidative stress may lead to renal ischemia caused by a systemic inflammatory response syndrome, so it seems logical that modulating the SIRS with antioxidants, especially selenium, can reduce the incidence of acute renal failure [44].

In the two above-mentioned outcomes (mortality and acute renal failure), although the number of studies was more than five (16 and 8 trials) and the effects estimated were considerable, the certainty of the evidence was low. Therefore, it is better to conduct high-quality RCTs to produce more definitive results and be more clinically practical.

Additionally, selenium supplementation revealed no substantial effect on 28-days mortality, adverse event, infection, and pneumonia, as well as the length of ICU and hospital stay and days on ventilation. In line with our findings, in the Cochrane Database Systematic Review by Allingstrup et al. [32], no clear evidence emerged for the benefits of selenium supplementation for outcomes such as 28-days mortality, adverse events, number of days on a ventilator, length of ICU stay, and length of hospital stay. An updated systematic review and meta-analysis showed that no useful effect of parenteral selenium was seen on the duration of hospital stay, days on the ventilator, and survival [41]. A systematic review and meta-analysis by Manzanares et al. [17] found that parenteral selenium as a single or combined therapy with other antioxidant micronutrients did not affect infections, length of ICU stay, length of hospital stay, or ventilator days.

In addition, in a recent meta-analysis, selenium administration decreased ICU and hospital stays, but did not affect infectious complications for patients who sustained major trauma [16].

Kong et al.’s meta-analysis [36] suggested that selenium administration was associated with significantly diminished length of hospital stay, but had no remarkable influence on 28-day mortality and length of ICU stay in septic patients. This is mainly because their analysis was based on only five RCTs and three of them had a relatively small sample size (*n* < 100), and the overestimation of the treatment effect was more likely in smaller trials compared with larger samples.

These contradictions in some results may be caused by differences in methods and duration of selenium administration, duration of treatment, and the number of included trials. Also, these could probably be due to factors that could not be readily incorporated into the protocol of studies, such as different timing of selenium supplementation and other aspects that could not be extracted from the papers.

Although supplementation with selenium is commonly considered well tolerated and safe in the majority of people, the high dose of selenium might cause toxicity due to its prooxidant features and, thus, should be used with caution [76]. Moreover, since selenium behaves dose dependently, it must be considered whether subjects have a normal or high level of selenium or are deficient. This might be due to the fact that the safety and efficacy of selenium might differ across levels of adequacy, deficiency, and toxicity [77]. For instance, Faghihi et al. [78] indicated that supplementation of selenium in participants with a normal range of selenium level caused an increase in selenium concentration (from 42.7 to 72 µg/l); as a result, subjects experienced adverse events of selenium therapy on glucose homeostasis.

Our study had several strengths. First, this is the first comprehensive evaluation and an overview of the current evidence on the effect of selenium supplementation on critical illness outcomes. Second, we were the first to use the AMSTAR2 and GRADE classification approaches to evaluate the quality and strength of the SRMAs evidence. Third, a low publication bias rate was detected among the included SRMAs. Fourth, though methodological designs were utilized correctly, selection bias may still exist, so two authors conducted these jobs with the strategies explained above to minimize this bias.

However, several limitations existed in our study. None of the included studies were classified as “high” quality according to the GRADE approach. A relatively large number of SRMAs were “Very low” and “Low” in GRADE categorizations, as well as “Critically Low” and “Low” in the AMSTAR2 classification. This phenomenon was mainly caused by many studies that failed to explain the included trials fully and did not register the protocol before conducting the meta-analysis. In all primary studies, there was a high risk of bias. There were only three RCTs reported for the outcome of pneumonia. There was a risk that some recently published RCTs were missed for this study since we included RCTs from SRMAs.

## Conclusion

According to our umbrella review, selenium supplementation in critically ill patients can reduce mortality and acute renal failure, and both reductions were statistically significant with the low certainty of evidence. Moreover, we found no significant efficacy of selenium supplementation on reducing the risks of 28-day mortality, adverse event, infection, and pneumonia, as well as the length of ICU and hospital stay and days on ventilation. Thus, due to the study limitations, general conclusions on the use of selenium supplementation for critically ill patients are not motivated.

## Supplementary Information

**Additional file 1: Table S1.** Search strategies including the key terms and the queries for each database. **Table S2.** List of excluded meta-analyses. **Table S3. **Methodological quality of included meta-analyses using AMSTAR2. **Table S4.** The GRADE quality of evidence for each outcome. **Table S5.** Subgroup analyses of selenium therapy on survival, infection, adverse events and acute renal failure. **Table S6.** Subgroup analyses of the effect of selenium therapy on length of hospital stay, length of intensive care unit stay and days on ventilation. **Figure S1.** The effect of Selenium therapy on the incidence of mortality (regardless of the period of time). **Figure S2.** The effect of Selenium therapy on the incidence of Mortality by duration (28 days). **Figure S3.** The effect of Selenium therapy on the risk of acute renal failure. **Figure S4.** The effect of Selenium therapy on the risk of infection. **Figure S5.** The effect of Selenium therapy on the risk of pneumonia. **Figure S6.** The effect of Selenium therapy on the length of intensive care unit (ICU) stay. **Figure S7.** The effect of Selenium therapy on the length of hospital stay.

## Data Availability

The data sets from this study are available from the corresponding author upon request.

## Abbreviations

APACHE score: Acute Physiology and Chronic Health Evaluation
AMSTAR2: A Measurement Tool to Assess Systematic Reviews
GRADE: Grading of Recommendations Assessment, Development, and Evaluation
ICU: Intensive care unit
MCID: Minimal clinically important difference
MD: Mean difference
RR: Risk ratio
OR: Odds ratio
PRIOR: Preferred Reporting Items for Overviews of Reviews
RCT: Randomized controlled trials
RoB: Risk of bias
ROS: Reactive oxygen species
SIRS: Systemic inflammatory response syndrome
SRMAs: Systematic reviews and meta-analyses
SOFA: Sequential Organ Failure Assessment
SAPS: Simplified Acute Physiology Score
SD: Standard deviation
