# Supplementary material for: The effect of selenium therapy in critically ill patients: an umbrella review of systematic reviews and meta-analysis of randomized controlled trials
Source: Eur J Med Res. 2023 Feb 28;28:104. doi: 10.1186/s40001-023-01075-w (PMC9972714; doi:10.1186/s40001-023-01075-w)
Supplement: Supplementary file 1 — Additional file 1: Table S1. Search strategies including the key terms and the queries for each database. Table S2. List of excluded meta-analyses. Table S3. Methodological quality of included meta-analyses using AMSTAR2. Table S4. The GRADE quality of evidence for each outcome. Table S5. Subgroup analyses of selenium therapy on survival, infection, adverse events and acute renal failure. Table S6. Subgroup analyses of the effect of selenium therapy on length of hospital stay, length of intensive care unit stay and days on ventilation. Figure S1. The effect of Selenium therapy on the incidence of mortality (regardless of the period of time). Figure S2. The effect of Selenium therapy on the incidence of Mortality by duration (28 days). Figure S3. The effect of Selenium therapy on the risk of acute renal failure. Figure S4. The effect of Selenium therapy on the risk of infection. Figure S5. The effect of Selenium therapy on the risk of pneumonia. Figure S6. The effect of Selenium therapy on the length of intensive care unit (ICU) stay. Figure S7. The effect of Selenium therapy on the length of hospital stay. [file 40001_2023_1075_MOESM1_ESM.docx]

**The effect of selenium therapy in critically ill patients: An umbrella review of systematic reviews and meta-analysis of randomized controlled trials**

Jaff et al.

Online Additional file

Additional data including 6 Additional Tables, 9 Additional Figures

| content | page |
| --- | --- |
| Table S1 | 2 |
| Table S2 | 3 |
| Table S3 | 4, 5 |
| Table S4 | 6-9 |
| Table S5 | 10,11 |
| Table S6 | 12 |
| Figure S1 | 13 |
| Figure S2 | 14 |
| Figure S3 | 15 |
| Figure S4 | 16 |
| Figure S5 | 17 |
| Figure S6 | 18 |
| Figure S7 | 19 |
| Figure S8 | 20 |
| Figure S9 | 21 |
| Additional References | 22 |

**Table S1.** Search strategies including the key terms and the queries for each database.

| **Database**  **8/22/2021** | **key terms and the queries** |
| --- | --- |
| PubMed  489 | #1 (((((((((((("meta analysis"[Title/Abstract]) OR ("meta-analyses"[Title/Abstract])) OR (metaanalysis[Title/Abstract])) OR ("meta-analyses"[Title/Abstract])) OR ("meta-analyze"[Title/Abstract])) OR ("meta-analysis"[Title/Abstract])) OR ("meta analysis"[Publication Type])) OR ("Systematic Review"[Title/Abstract])) OR ("Systematic Review"[Publication Type])) OR ("Systematic Reviews as Topic"[MeSH Terms])) OR ("meta analysis"[Publication Type])) OR ("Meta-Analysis as Topic"[MeSH Terms]))  #2 (("Selenium"[Mesh]) OR (((((((selenium[Title/Abstract]) OR ("selenious acid"[Title/Abstract])) OR ("sodium selenious acid"[Title/Abstract])) OR ("sodium selenite"[Title/Abstract])) OR ("antioxidant cocktails"[Title/Abstract])) OR ("selenium derivative"[Title/Abstract])) OR ("selenium compounds"[Title/Abstract])))  #3 #1 AND #2 |
| Web of Science (ISI)  716 | 1# **TOPIC: (Selenium) OR TOPIC: ("selenious acid") OR TOPIC: ("sodium selenious acid") OR TOPIC: ("sodium selenite") OR TOPIC: ("antioxidant cocktails") OR TOPIC: ("selenium compounds")**  #2  **TOPIC:** ("oral cooling") *OR* **TOPIC:** (cryotherapy)  #3 #1 AND #2 |
| Scopus  1247 | #1 ( ( TITLE-ABS-KEY ( "meta analysis" ) OR TITLE-ABS-KEY ( "meta-analyses" ) OR TITLE-ABS-KEY ( "meta-analysis" ) OR TITLE-ABS-KEY ( "meta-analyses" ) OR TITLE-ABS-KEY ( "meta-analyze" ) OR TITLE-ABS-KEY ( "metaanalysis" ) OR TITLE-ABS-KEY ( "Systematic Review" ) OR TITLE-ABS-KEY ( "Systematic Reviews as Topic" ) OR TITLE-ABS-KEY ( "Meta-Analysis as Topic" ) ) )  #2 ( ( TITLE-ABS-KEY ( selenium ) OR TITLE-ABS-KEY ( "selenious acid" ) OR TITLE-ABS-KEY ( "sodium selenious acid" ) OR TITLE-ABS-KEY ( "sodium selenite" ) OR TITLE-ABS-KEY ( "antioxidant cocktails" ) OR TITLE-ABS-KEY ( "selenium derivative" ) OR TITLE-ABS-KEY ( "selenium compounds" ) ) )  #3 #1 AND #2 |

**Search: 2452 7/10/2022 Duplicate: 805**

**Table S2.** List of excluded meta-analyses^1^.

| Network meta-analysis (n=1) (1) |
| --- |
| Not interested intervention (n=1) (2) |
| Systematic review without meta-analysis (n=1) (3) |
| ^1^ Numbers in parenthesis refer to supplemental references. |

**Table S3.** Methodological quality of included meta-analyses using AMSTAR2

| **Author, year (ref)** | **Q1** | **Q2** | **Q3** | **Q4** | **Q5** | **Q6** | **Q7** | **Q8** | **Q9** | **Q10** | **Q11** | **Q12** | **Q13** | **Q14** | **Q15** | **Q16** | **Overall quality** |
| --- | --- | --- | --- | --- | --- | --- | --- | --- | --- | --- | --- | --- | --- | --- | --- | --- | --- |
| Allingstrup, 2015 (4) | Yes | Yes | Yes | PY | Yes | Yes | Yes | Yes | Yes | Yes | Yes | Yes | Yes | Yes | Yes | Yes | High |
| Alhazzani, 2013 (5) | Yes | No | Yes | PY | Yes | Yes | Yes | No | Yes | Yes | Yes | Yes | Yes | Yes | Yes | Yes | Low |
| Avenell, 2010 (6) | Yes | Yes | Yes | PY | Yes | Yes | Yes | Yes | Yes | Yes | Yes | Yes | Yes | Yes | Yes | Yes | High |
| Huang, 2022 (7) | Yes | Yes | Yes | PY | Yes | Yes | PY | Yes | Yes | Yes | Yes | No | No | Yes | Yes | Yes | Low |
| He, 2020 (8) | Yes | No | Yes | PY | Yes | Yes | No | No | Yes | No | Yes | Yes | Yes | Yes | Yes | No | Critically low |
| Huang, 2012 (9) | Yes | PY | Yes | PY | Yes | Yes | Yes | Yes | Yes | Yes | Yes | Yes | Yes | Yes | Yes | Yes | High |
| Heyland, 2005 (10) | Yes | PY | Yes | PY | Yes | Yes | Yes | No | Yes | No | Yes | Yes | Yes | Yes | Yes | No | Moderate |
| Kong, 2021 (11) | Yes | PY | Yes | PY | Yes | Yes | Yes | No | Yes | Yes | Yes | No | No | Yes | Yes | Yes | Low |
| Kong, 2013 (12) | Yes | PY | Yes | PY | Yes | Yes | PY | Yes | Yes | Yes | Yes | Yes | No | Yes | Yes | Yes | Low |
| Li, 2019 (13) | Yes | Yes | Yes | PY | Yes | Yes | No | Yes | Yes | Yes | Yes | Yes | Yes | Yes | Yes | Yes | Low |
| Landucci, 2013 (14) | Yes | No | Yes | PY | Yes | Yes | Yes | Yes | Yes | No | Yes | Yes | Yes | Yes | No | No | Critically low |
| Mousavi, 2021 (15) | Yes | No | Yes | PY | Yes | Yes | PY | Yes | Yes | Yes | Yes | No | No | Yes | Yes | Yes | Critically low |
| Manzanares, 2016 (16) | Yes | No | Yes | PY | Yes | Yes | Yes | No | Yes | Yes | Yes | Yes | Yes | Yes | Yes | Yes | Low |
| Manzanares, 2012 (17) | Yes | No | Yes | PY | Yes | Yes | Yes | No | Yes | No | Yes | No | No | Yes | Yes | Yes | Critically low |
| Miller, 2013 (18) | Yes | No | Yes | PY | Yes | Yes | Yes | No | Yes | No | Yes | Yes | Yes | Yes | No | No | Critically low |
| Visser, 2011 (19) | Yes | No | Yes | PY | Yes | Yes | Yes | No | Yes | No | Yes | Yes | No | Yes | Yes | No | Critically low |
| Zhao, 2019 (20) | Yes | Yes | Yes | PY | Yes | Yes | PY | Yes | Yes | Yes | Yes | Yes | Yes | Yes | Yes | Yes | High |

Ref, references; PY, partially yes. **Q1:** Did the research questions and inclusion criteria for the review include the components of PICO?, **Q2**: 2. Did the report of the review contain an explicit statement that the review methods were established prior to the conduct of the review and did the report justify any significant deviations from the protocol?; **Q3**, Did the review authors explain their selection of the study designs for inclusion in the review?; **Q4**, Did the review authors use a comprehensive literature search strategy?; **Q5**, Did the review authors perform study selection in duplicate?; **Q6**, Did the review authors perform data extraction in duplicate?; **Q7**, Did the review authors provide a list of excluded studies and justify the exclusions?; **Q8**, Did the review authors describe the included studies in adequate detail?; **Q9**, Did the review authors use a satisfactory technique for assessing the risk of bias?; **Q10**, Did the review authors report on the sources of funding?; **Q11**, Did the review authors use appropriate methods for statistical combination of results?; **Q12**, Did the review authors assess the potential impact of RoB in individual studies on the results?; **Q13**, Did the review authors account for RoB in individual studies when interpreting/ discussing the results of the review?; **Q14**, Did the review authors provide a satisfactory explanation for, and discussion of, any heterogeneity?; **Q15**, Did the review authors carry out an adequate investigation of publication bias?; **Q16**, Did the review authors report any potential sources of conflict of interest?

**Table S4.** The GRADE quality of evidence for each outcome**.**

| **Certainty assessment** | | | | | | | **№ of patients** | | **Effect** | | **Certainty** | **Importance** |
| --- | --- | --- | --- | --- | --- | --- | --- | --- | --- | --- | --- | --- |
| **№ of studies** | **Study design** | **Risk of bias** | **Inconsistency** | **Indirectness** | **Imprecision** | **Other considerations** | **selenium** | **any control** | **Relative (95% CI)** | **Absolute (95% CI)** |  |  |
|  |  |  |  |  |  |  |  |  |  |  |  |  |
| **Mortality** | | | | | | | | | | | | |
| 16 | randomised trials | serious^a^ | not serious^b^ | not serious | not serious^c^ | publication bias strongly suspected^d^ | 352/1161 (30.3%) | 392/1163 (33.7%) | **RR 0.83** (0.71 to 0.98) | **57 fewer per 1,000** (from 98 fewer to 7 fewer) | ⨁◯◯◯ Low | CRITICAL |
| **Mortality by duration (28 days)** | | | | | | | | | | | | |
| 21 | randomised trials | serious^e^ | not serious^f^ | not serious | not serious^g^ | none | 488/1839 (26.5%) | 544/1875 (29.0%) | **RR 0.95** (0.82 to 1.09) | **15 fewer per 1,000** (from 52 fewer to 26 more) | ⨁⨁⨁◯ Moderate | CRITICAL |
| **Risk of infection** | | | | | | | | | | | | |
| 9 | randomised trials | serious^h^ | not serious^i^ | not serious | not serious^j^ | none | 496/1037 (47.8%) | 519/1046 (49.6%) | **RR 0.92** (0.80 to 1.05) | **40 fewer per 1,000** (from 99 fewer to 25 more) | ⨁⨁⨁◯ Moderate | CRITICAL |
| **Adverse events** | | | | | | | | | | | | |
| 7 | randomised trials | serious^k^ | not serious^l^ | not serious | not serious^m^ | none | 358/1002 (35.7%) | 359/1012 (35.5%) | **RR 0.98** (0.88 to 1.10) | **7 fewer per 1,000** (from 43 fewer to 35 more) | ⨁⨁⨁◯ Moderate | IMPORTANT |
| **Risk of pneumonia** | | | | | | | | | | | | |
| 3 | randomised trials | serious^n^ | not serious^o^ | not serious | serious^p^ | none | 27/138 (19.6%) | 26/142 (18.3%) | **RR 1.11** (0.72 to 1.72) | **20 more per 1,000** (from 51 fewer to 132 more) | ⨁⨁◯◯ Low | CRITICAL |
| **Risk of acute renal failure** | | | | | | | | | | | | |
| 8 | randomised trials | serious^q^ | not serious^r^ | not serious | serious^s^ | none | 40/351 (11.4%) | 62/349 (17.8%) | **RR 0.67** (0.46 to 0.98) | **59 fewer per 1,000** (from 96 fewer to 4 fewer) | ⨁⨁◯◯ Low | CRITICAL |
| **Length of ICU stay** | | | | | | | | | | | | |
| 13 | randomised trials | serious^t^ | serious^u^ | not serious | serious^v^ | none | 1431 | 1497 | - | MD **0.15 Day higher** (1.75 lower to 2.05 higher) | ⨁◯◯◯ Very low | IMPORTANT |
| **Length of hospital stay** | | | | | | | | | | | | |
| 9 | randomised trials | serious^w^ | serious^x^ | not serious | serious^y^ | none | 774 | 823 | - | MD **0.51 day lower** (3.74 lower to 2.72 higher) | ⨁◯◯◯ Very low | IMPORTANT |
| **Days on ventilation** | | | | | | | | | | | | |
| 8 | randomised trials | serious^z^ | serious^aa^ | not serious | serious^ab^ | none | 213 | 213 | - | MD **0.98 day lower** (2.93 lower to 0.98 higher) | ⨁◯◯◯ Very low | IMPORTANT |

CI: confidence interval; MD: mean difference; RR: risk ratio

**Explanations**

a. Serious risk of bias since 14 studies were at high risk of bias. Downgraded.

b. Not serious inconsistency since I2 = 31.9%, Phet =0.110. Not downgraded.

c. Optimal information size met. The 95%CI excluded the null value (RR: 1.00). Not downgraded.

d. Serious publication bias since P Egger = 0.003 and there was asymmetry in the funnel plot. Downgraded.

e. Serious risk of bias since 17 studies were at high risk of bias. Downgraded.

f. Not serious inconsistency since I2 = 21.1%, Phet =0.189. Not downgraded.

g. Optimal information size met. The 95%CI included the null value (RR: 1.00), but the lower and upper bound of the 95%CI were >0.75 and <1.25. Not downgraded.

h. Serious risk of bias since eight studies were at high risk of bias. Downgraded.

i. Not serious inconsistency since I2 = 0%, Phet =0.620. Not downgraded.

j. Optimal information size met. The 95%CI included the null value (RR: 1.00), but the lower and upper bound of the 95%CI were >0.75 and <1.25. Not downgraded.

k. Serious risk of bias since six studies were at high risk of bias. Downgraded.

l. Not serious inconsistency since I2 = 26.4%, Phet =0.227. Not downgraded.

m. Optimal information size met. The 95%CI included the null value (RR: 1.00), but the lower and upper bound of the 95%CI were >0.75 and <1.25. Not downgraded.

n. Serious risk of bias since three studies were at high risk of bias. Downgraded.

o. Not serious inconsistency since I2 = 5.1%, Phet =0.349. Not downgraded.

p. Optimal information size did not meet. The 95%CI included the null value (RR: 1.00) and the upper bound of the 95%CI >1.25. Downgraded.

q. Serious risk of bias since six studies were at high risk of bias. Downgraded.

r. Not serious inconsistency since I2 = 0%, Phet =0.659. Not downgraded.

s. Optimal information size did not meet. The 95%CI included the null value (RR: 1.00) and the lower bound of the 95%CI <0.75. Downgraded.

t. Serious risk of bias since 11 studies were at high risk of bias. Downgraded.

u. Serious inconsistency since I2 = 95.8%, Phet <0.001. Downgraded.

v. Optimal information size met. The effect size (WMD: 0.15) did not surpass the minimal clinically important difference for the length of ICU stay (MCID: 4.83 days). Downgraded.

w. Serious risk of bias since eight studies were at high risk of bias. Downgraded.

x. Serious inconsistency since I2 = 96%, Phet <0.001. Downgraded.

y. Optimal information size met. The effect size (WMD: -0.51) did not surpass the minimal clinically important difference for the length of hospital stay (MCID: -6.11 days). Downgraded.

z. Serious risk of bias since six studies were at high risk of bias. Downgraded.

aa. Serious inconsistency since I2 = 76.4%, Phet <0.001. Downgraded.

ab. Optimal information size did not meet. The effect size (WMD: -0.98) did not surpass the minimal clinically important difference for the days on ventilation (MCID: -5.6 days). Downgraded.

**Table S5.** Subgroup analyses of selenium therapy on survival, infection, adverse events and acute renal failure.

| **Sub-grouped by** | **No. of trials** | **Effect size^1^** | **95% CI** | **I^2^ (%)** | **P for heterogeneity^2^** | **P for between**  **subgroup heterogeneity^3^** |
| --- | --- | --- | --- | --- | --- | --- |
| **Mortality (regardless of period of time)** | | | | | | |
| First dose (µg/d) | | | | | | 0.592 |
| ≤1000 | 12 | 0.84 | 0.70, 1.02 | 36.5% | 0.098 |  |
| >1000 | 4 | 0.78 | 0.58, 1.04 | 12.2% | 0.332 |  |
| Following dose (µg/d) | | | | | | 0.169 |
| ≤1000 | 13 | 0.88 | 0.73, 1.04 | 31.7% | 0.129 |  |
| >1000 | 3 | 0.83 | 0.71, 0.98 | 0.0% | 0.679 |  |
| Selenium in the control group (low dose) | | | | | | 0.550 |
| Yes | 4 | 0.94 | 0.72, 1.22 | 0.0% | 0.675 |  |
| No | 11 | 0.79 | 0.63, 0.99 | 49.7% | 0.030 |  |
| Duration of intervention (days) | | | | | | 0.700 |
| ≤10 | 7 | 0.87 | 0.71, 1.05 | 0.0% | 0.621 |  |
| >10 | 7 | 0.74 | 0.54, 1.01 | 63.8 % | 0.011 |  |
| **Mortality by duration (28 days)** | | | | | | |
| First dose (µg/d) | | | | | | 0.987 |
| ≤1000 | 16 | 0.94 | 0.79, 1.12 | 37.8% | 0.063 |  |
| >1000 | 5 | 0.95 | 0.65, 1.39 | 0.0% | 0.784 |  |
| Following dose (µg/d) | | | | | | 0.580 |
| ≤1000 | 17 | 0.96 | 0.81, 1.13 | 31.8% | 0.102 |  |
| >1000 | 4 | 0.84 | 0.57, 1.24 | 0.0% | 0.733 |  |
| Selenium in the control group (low dose) | | | | | | 0.076 |
| Yes | 7 | 1.08 | 0.93, 1.24 | 0.0% | 0.593 |  |
| No | 13 | 0.87 | 0.69, 1.09 | 24.5% | 0.197 |  |
| Duration of intervention (days) | | | | | | 0.368 |
| ≤10 | 9 | 1.03 | 0.84, 1.23 | 11.3% | 0.340 |  |
| >10 | 11 | 0.87 | 0.69, 1.10 | 36.5% | 0.107 |  |
| **Risk of acute renal failure** | | | | | | |
| First dose (µg/d) | | | | | | 0.292 |
| ≤1000 | 3 | 0.46 | 0.20, 1.04 | 30.0% | 0.240 |  |
| >1000 | 5 | 0.78 | 0.50, 1.22 | 0.0% | 0.936 |  |
| Following dose (µg/d) | | | | | | 0.373 |
| ≤1000 | 5 | 0.57 | 0.35, 0.94 | 0.0% | 0.458 |  |
| >1000 | 3 | 0.83 | 0.47, 1.46 | 0.0% | 0.800 |  |
| Selenium in the control group (low dose) | | | | | | 0.878 |
| Yes | 3 | 0.61 | 0.26, 1.47 | 21.5% | 0.280 |  |
| No | 5 | 0.68 | 0.44, 1.06 | 0.0% | 0.657 |  |
| Duration of intervention (days) | | | | | | 0.733 |
| ≤10 | 5 | 0.58 | 0.31, 1.06 | 11.6% | 0.339 |  |
| >10 | 2 | 0.69 | 0.37, 1.27 | 0.0% | 0.796 |  |
| **Adverse events** | | | | | | |
| First dose (µg/d) | | | | | | 0.099 |
| ≤1000 | 5 | 0.94 | 0.88, 1.00 | 0.0% | 0.468 |  |
| >1000 | 2 | 1.19 | 0.95, 1.49 | 0.0% | 0.489 |  |
| Following dose (µg/d) | | | | | | 0.437 |
| ≤1000 | 6 | 0.97 | 0.85, 1.11 | 29.9% | 0.211 |  |
| >1000 | 1 | 1.11 | 0.82, 1.50 | - | - |  |
| Selenium in the control group (low dose) | | | | | | 0.729 |
| Yes | 2 | 1.08 | 0.64, 1.82 | 0.0% | 0.604 |  |
| No | 5 | 0.99 | 0.86, 1.14 | 47.8% | 0.105 |  |
| Duration of intervention (days) | | | | | | 0.180 |
| ≤10 | 4 | 1.11 | 0.82. 1.49 | 38.5% | 0.181 |  |
| >10 | 2 | 0.94 | 0.88, 1.00 | 0.0% | 0.914 |  |
| **Risk of infection** | | | | | | |
| First dose (µg/d) | | | | | | 0.764 |
| ≤1000 | 4 | 0.91 | 0.79, 1.05 | 0.0% | 0.815 |  |
| >1000 | 5 | 0.90 | 0.52, 1.57 | 23.1% | 0.267 |  |
| Following dose (µg/d) | | | | | | 0.251 |
| ≤1000 | 6 | 0.93 | 0.83, 1.07 | 0.0% | 0.654 |  |
| >1000 | 3 | 0.59 | 0.29, 1.19 | 0.0% | 0.503 |  |
| Selenium in the control group (low dose) | | | | | | 0.891 |
| Yes | 2 | 0.91 | 0.77, 1.07 | 0.0% | 0.919 |  |
| No | 7 | 0.93 | 0.73, 1.20 | 3.4% | 0.400 |  |
| Duration of intervention (days) | | | | | | 0.763 |
| ≤10 | 6 | 0.92 | 0.80, 1.07 | 0.0% | 0.450 |  |
| >10 | 2 | 0.87 | 0.64, 1.18 | 0.0% | 0.691 |  |

CI, confidence interval

^1^Calculated by Random-effects model

^2^P heterogeneity within subgroup ^3^P heterogeneity between subgroups using meta-regression analysis

**Table S6.** Subgroup analyses of the effect of selenium therapy on length of hospital stay, length of intensive care unit stay and days on ventilation.

| **Sub-grouped by** | **No. of trials** | **Effect size^1^** | **95% CI** | **I^2^ (%)** | **P for heterogeneity^2^** | **P for between**  **subgroup heterogeneity^3^** |
| --- | --- | --- | --- | --- | --- | --- |
| **Length of hospital stay** | | | | | | |
| First dose (µg/d) | | | | | | 0.801 |
| ≤1000 | 5 | -0.83 | -5.77, 4.10 | 97.9% | <0.001 |  |
| >1000 | 4 | -0.98 | -2.73, 0.77 | 12.1% | 0.332 |  |
| Following dose (µg/d) | | | | | | 0.447 |
| ≤1000 | 7 | -1.02 | -4.75, 2.72 | 96.9% | <0.001 |  |
| >1000 | 2 | 1.18 | -1.86, 4,22 | 0.0% | 0.705 |  |
| Selenium in the control group (low dose) | | | | | | 0.187 |
| Yes | 2 | -3.13 | -6.75, 0.49 | 69.6% | 0.070 |  |
| No | 7 | 0.27 | -2.75, 3.30 | 88.5% | <0.001 |  |
| Duration of intervention (days) | | | | | | 0.891 |
| ≤10 | 6 | -0.38 | -5.01, 4.25 | 97.4% | <0.001 |  |
| >10 | 3 | -1.48 | -2.91, -0.05 | 0.0% | 0.423 |  |
| **Length of ICU stay** | | | | | | |
| First dose (µg/d) | | | | | | 0.832 |
| ≤1000 | 8 | 0.22 | -2.53, 2.96 | 97.4% | <0.001 |  |
| >1000 | 5 | -0.08 | -2.72, 2.56 | 51.9% | 0.081 |  |
| Following dose (µg/d) | | | | | | 0.287 |
| ≤1000 | 10 | 0.59 | -1.49, 2.68 | 96.8% | <0.001 |  |
| >1000 | 3 | -2.10 | -5.50, 1.29 | 0.0% | 0.483 |  |
| Selenium in the control group (low dose) | | | | | | 0.224 |
| Yes | 3 | -1.72 | -2.49, -0.95 | 0.0% | 0.836 |  |
| No | 10 | 0.64 | -1.56, 2.85 | 96.4% | <0.001 |  |
| Duration of intervention (days) | | | | | | 0.468 |
| ≤10 | 7 | 0.60 | 3.02, 4.23 | 96.5% | <0.001 |  |
| >10 | 5 | -0.53 | -1.37, 0.32 | 57.2% | 0.053 |  |
| **Days on ventilation** | | | | | | |
| First dose (µg/d) | | | | | | 0.174 |
| ≤1000 | 4 | -2.54 | -4.24, -0.84 | 49.5% | 0.114 |  |
| >1000 | 4 | 0.64 | -2.83, 4.11 | 75.8% | 0.008 |  |
| Following dose (µg/d) | | | | | | 0.988 |
| ≤1000 | 5 | -0.89 | -3.80, 2.02 | 85.4% | <0.001 |  |
| >1000 | 3 | -1.23 | -3.18, 0.73 | 0.0% | 0.542 |  |
| Selenium in the control group (low dose) | | | | | | 0.513 |
| Yes | 1 | -2.70 | -3.49, -1.91 | - | - |  |
| No | 7 | -0.56 | -3.10, 1.98 | 75.1% | 0.001 |  |
| Duration of intervention (days) | | | | | | 0.827 |
| ≤10 | 6 | -0.75 | -3.43, 1.92 | 82.7% | <0.001 |  |
| >10 | 2 | -1.48 | -3.56, 0.59 | 0.0% | 0.676 |  |

ICU, intensive care unit; CI, confidence interval.

^1^Calculated by Random-effects model

^2^P heterogeneity within subgroup ^3^P heterogeneity between subgroups using meta-regression analysis

**
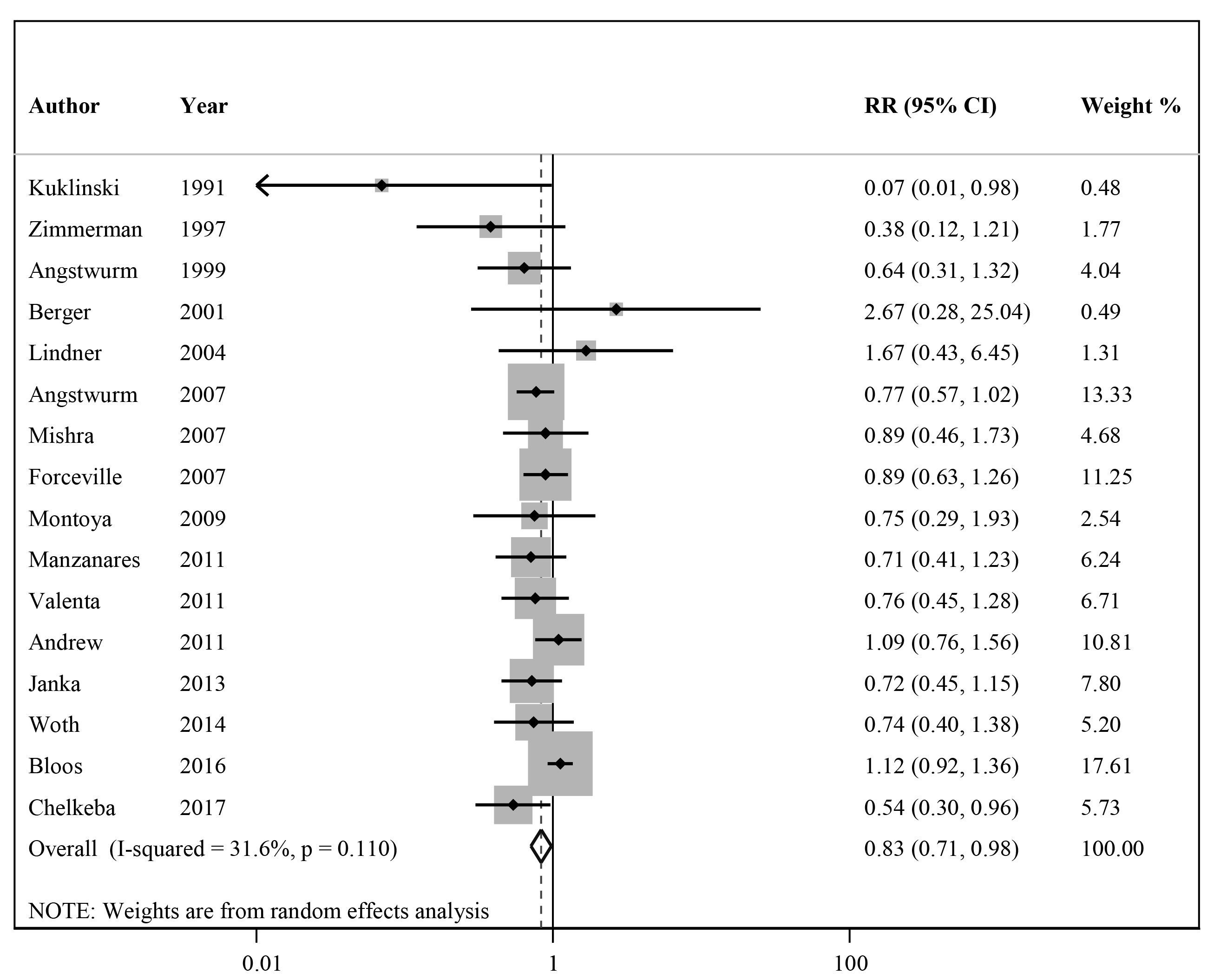
**

**Figure S1.** The effect of Selenium therapy on the incidence of mortality (regardless of the period of time)

.





**Figure S2.** The effect of Selenium therapy on the incidence of Mortality by duration (28 days)





**Figure S3.** The effect of Selenium therapy on the risk of acute renal failure

**

**

**Figure S4.** The effect of Selenium therapy on the risk of infection.

**

**

**Figure S5.** The effect of Selenium therapy on the risk of pneumonia.





**Figure S6.** The effect of Selenium therapy on the length of intensive care unit (ICU) stay





**Figure S7.** The effect of Selenium therapy on the length of hospital stay





**Figure S8.** The effect of Selenium therapy on the days on ventilation.

**

**

**Figure S9.** The effect of Selenium therapy on adverse events incidence.

1. Gudivada KK, Kumar A, Sriram K, Baby J, Shariff M, Sampath S, et al. Antioxidant micronutrient supplements for adult critically ill patients: A bayesian multiple treatment comparisons meta-analysis. Clinical nutrition ESPEN. 2022;47:78-88.

2. Kurmis R, Greenwood J, Aromataris E. Trace Element Supplementation Following Severe Burn Injury: A Systematic Review and Meta-Analysis. Journal of burn care & research : official publication of the American Burn Association. 2016;37(3):143-59.

3. Adjepong M, Agbenorku P, Brown P, Oduro I. The role of antioxidant micronutrients in the rate of recovery of burn patients: a systematic review. Burns & trauma. 2016;4:18.

4. Allingstrup M, Afshari A. Selenium supplementation for critically ill adults. The Cochrane database of systematic reviews. 2015;2015(7):Cd003703.

5. Alhazzani W, Jacobi J, Sindi A, Hartog C, Reinhart K, Kokkoris S, et al. The effect of selenium therapy on mortality in patients with sepsis syndrome: a systematic review and meta-analysis of randomized controlled trials. Critical care medicine. 2013;41(6):1555-64.

6. Avenell A, Noble DW, Barr J, Engelhardt T. Selenium supplementation for critically ill adults. The Cochrane database of systematic reviews. 2004(4):Cd003703.

7. Huang JF, Hsu CP, Ouyang CH, Cheng CT, Wang CC, Liao CH, et al. The Impact of Selenium Supplementation on Trauma Patients-Systematic Review and Meta-Analysis. Nutrients. 2022;14(2).

8. He S, Cao B, Yu H, Zhen J, Wang F. REPORT-Parenteral selenium for the clinical effect of sepsis: A meta-analysis of randomized controlled trials. Pakistan journal of pharmaceutical sciences. 2020;33(6):2679-86.

9. Huang TS, Shyu YC, Chen HY, Lin LM, Lo CY, Yuan SS, et al. Effect of parenteral selenium supplementation in critically ill patients: a systematic review and meta-analysis. PloS one. 2013;8(1):e54431.

10. Heyland DK, Dhaliwal R, Suchner U, Berger MM. Antioxidant nutrients: a systematic review of trace elements and vitamins in the critically ill patient. Intensive care medicine. 2005;31(3):327-37.

11. Kong L, Wu Q, Liu B. The impact of selenium administration on severe sepsis or septic shock: a meta-analysis of randomized controlled trials. African health sciences. 2021;21(1):277-85.

12. Kong Z, Wang F, Ji S, Deng X, Xia Z. Selenium supplementation for sepsis: a meta-analysis of randomized controlled trials. The American journal of emergency medicine. 2013;31(8):1170-5.

13. Li S, Tang T, Guo P, Zou Q, Ao X, Hu L, et al. A meta-analysis of randomized controlled trials: Efficacy of selenium treatment for sepsis. Medicine. 2019;98(9):e14733.

14. Landucci F, Mancinelli P, De Gaudio AR, Virgili G. Selenium supplementation in critically ill patients: a systematic review and meta-analysis. Journal of critical care. 2014;29(1):150-6.

15. Mousavi MA, Saghaleini SH, Mahmoodpoor A, Ghojazadeh M, Mousavi SN. Daily parenteral selenium therapy in critically ill patients: An updated systematic review and meta-analysis of randomized controlled trials. Clinical nutrition ESPEN. 2021;41:49-58.

16. Manzanares W, Lemieux M, Elke G, Langlois PL, Bloos F, Heyland DK. High-dose intravenous selenium does not improve clinical outcomes in the critically ill: a systematic review and meta-analysis. Critical care (London, England). 2016;20(1):356.

17. Manzanares W, Dhaliwal R, Jiang X, Murch L, Heyland DK. Antioxidant micronutrients in the critically ill: a systematic review and meta-analysis. Critical care (London, England). 2012;16(2):R66.

18. Miller N, Miller M, Hill LT. The impact of antioxidant supplementation on clinical outcomes in the critically ill: A meta-analysis. Southern African Journal of Critical Care. 2013;29(1):18-26.

19. Visser J, Labadarios D, Blaauw R. Micronutrient supplementation for critically ill adults: a systematic review and meta-analysis. Nutrition (Burbank, Los Angeles County, Calif). 2011;27(7-8):745-58.

20. Zhao Y, Yang M, Mao Z, Yuan R, Wang L, Hu X, et al. The clinical outcomes of selenium supplementation on critically ill patients: A meta-analysis of randomized controlled trials. Medicine. 2019;98(20):e15473.

**References**
